# Chromosome-level genome assembly of the intertidal lucinid clam *Indoaustriella scarlatoi*

**DOI:** 10.1038/s41597-025-04606-8

**Published:** 2025-02-15

**Authors:** Yang Guo, Zhaoshan Zhong, Nannan Zhang, Minxiao Wang, Chaolun Li

**Affiliations:** 1 Center of Deep-sea Research, Institute of Oceanology, Chinese Academy of Sciences, Qingdao, 266071 China; 2 State Key Laboratory of Microbial Technology, Shandong University, Qingdao, Shandong 266237 China; 3 BGI Research, Sanya, 572025 China; 4 Hainan Technology Innovation Center for Marine Biological Resources Utilization (Preparatory Period), BGI Research, Sanya, 572025 China; 5 South China Sea Institute of Oceanology, Chinese Academy of Sciences, Guangzhou, 510301 China

**Keywords:** Evolutionary genetics, Ecology

## Abstract

Lucinidae, renowned as the most diverse chemosymbiotic invertebrate group, functions as a sulfide cleaner in coastal ecosystems and is thus ecologically important. Despite their significance, genomic studies on these organisms have been limited. Here, we present the chromosome-level genome assembly of *Indoaustriella scarlatoi*, an intertidal lucinid clam. Employing both short and long reads, and Hi-C sequencing, we assembled a 1.58 Gb genome comprising 690 contigs with a contig N50 length of 9.00 Mb, which were anchored to 17 chromosomes. The genome exhibits a high completeness of 95.4%, as assessed by the BUSCO analysis. Transposable elements account for 56.02% of the genome, with long terminal repeat retrotransposons (LTR, 42.66%) being the most abundant. We identified 34,469 protein-coding genes, 74.43% of which were functionally annotated. This high-quality genome assembly serves as a valuable resource for further studies on the evolutionary and ecological aspects of chemosymbiotic bivalves.

## Background & Summary

Lucinidae (Bivalvia: Lucinida) is the most species-rich family of chemosymbiotic invertebrates^1^. All known species of this bivalve family have established symbiotic relationships with chemosynthetic *Gammaproteobacteria*^2,3^. Lucinids are widely distributed in marine ecosystems, ranging from 70° N to 55° S, including intertidal zones, shallow-water, and deep-sea sediments^4^. Previous studies have demonstrated the evolution of deep-sea bivalves to chemosymbiosis^5–7^, but coastal ones may have different adaptations due to the higher availability of photosynthetic matter in coastal ecosystems than the deep-sea habitats. However, the specific evolutionary adaptations of coastal bivalves to chemosymbiosis remain largely unknown. Furthermore, Lucinidae and Thyasiridae (Lucinida) have long been considered as closely related groups due to the shared morphological features. However, phylogenetic trees based on rRNA genes supported the monophyletic status of each group^8^, and genomic studies of both Thyasiridae^7^ and Lucinidae species will further promote the understanding of these questions.

Lucinids have been proved to play a pivotal ecological role in coastal ecosystems. Through large-scale genomic studies, coastal lucinid symbionts mainly belong to the genus *Ca. Thiodiazotropha* and are universally capable of sulfur oxidation and carbon fixation^9,10^, enabling the lucinid holobionts to effectively remove sulfides from sediment. The presence of lucinid clams significantly reduces the concentration of sulfides in sediment, as demonstrated in either mesocosm or field experiments^11–13^. This process is crucial for maintaining the health of plants in coastal areas, as high levels of hydrogen sulfide can severely affect the development of the roots of seagrasses and mangroves^11,14^. Therefore, lucinids and their bacterial symbionts are of great ecological importance in coastal ecosystems^11^.

**Fig. 1:**
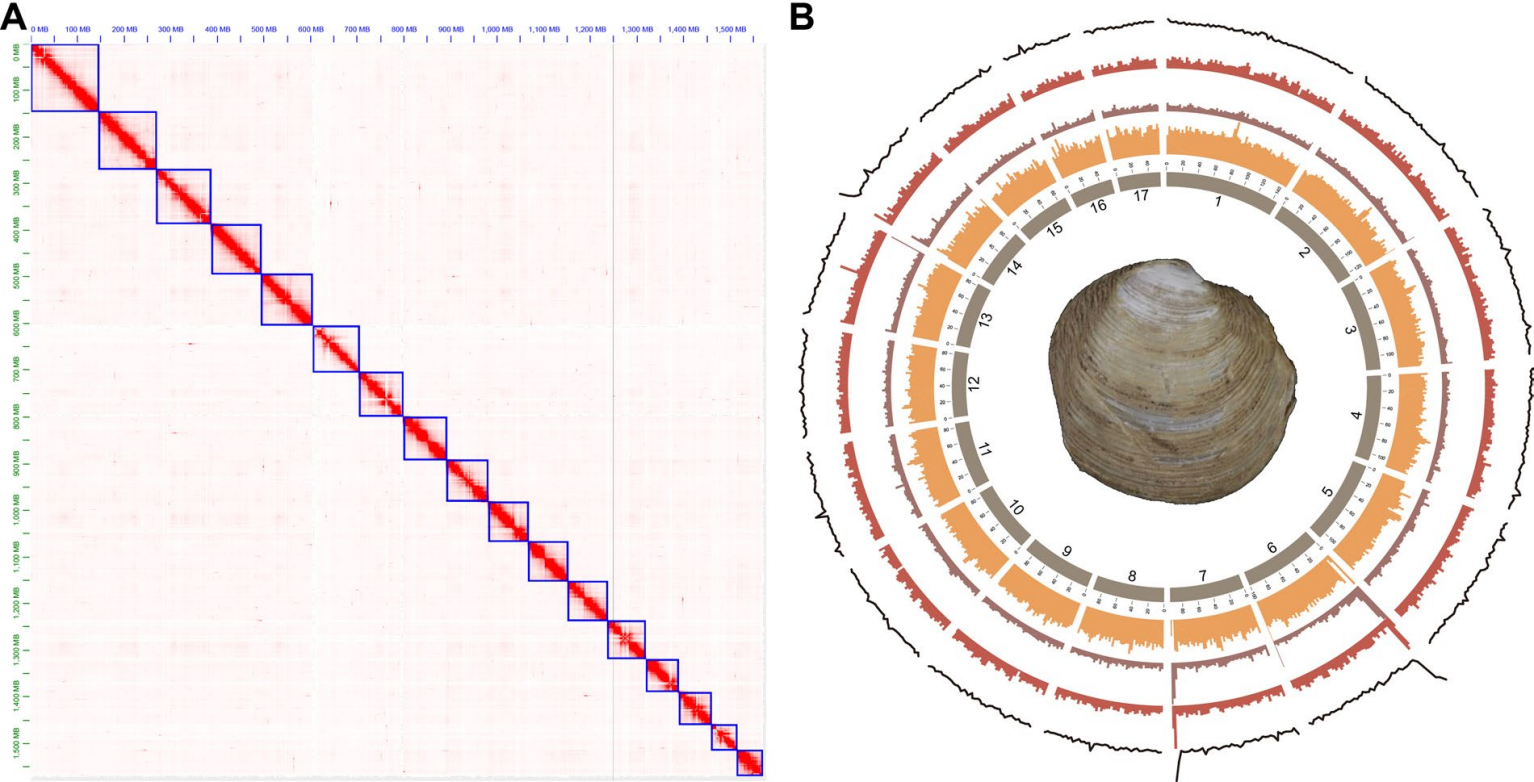
Genomic characteristics of *Indoaustriella scarlatoi*. (**A**) Genome-wide all-by-all Hi-C matrix. (**B**) Circos view of the assembled chromosomes showing marker distributions at 2-Mb sliding windows from outer to inner circle: GC content, gene density, tandem repeat density, transposable element density.

Despite lucinids’ significant importance in the fields of evolution and ecology, the lack of genomic data has hindered the study of their phylogenetic relationships, evolutionary adaptations, and the regulatory mechanisms behind their ecological functions. Here, we assembled the chromosome-level genome of *Indoaustriella scarlatoi* (Lucinidae) based on reads of whole genome sequencing (WGS), PacBio HiFi sequencing, and Hi-C sequencing (Table 1). The *I. scarlatoi* genome is 1.58 Gb in size, containing 690 contigs with a N50 length of 9.00 Mb (Table 2). After Hi-C scaffolding, 99.41% of contigs were anchored to 17 chromosomes with a scaffold N50 length of 94.81 Mb (Tables 2, 3, Fig. 1). The mapping rate of WGS reads is 98.15%. In total, 938 genes, including 911 complete ones, of the 954 metazoan Benchmarking Universal Single-Copy Orthologs (BUSCO) were successfully located in the final assembly, indicating that the genome completeness is 95.4% (Table 2). The transposable elements occupied 56.02% of the genome, while LTR accounted for 42.66% of the genome (Table 4). We predicted 34,469 protein-coding genes in the *I. scarlatoi* genome, and 74.43% of these genes can be functionally annotated using at least one public database (Table 5). The ncRNA including tRNA, rRNA, miRNA, and snRNA were annotated with a total length of 1.35 Mb (Table 6). Overall, the *I. scarlatoi* genome is of high quality and will provide a valuable resource for studies on phylogeny and adaptive evolution.

**Table 1:** Statistics of sequencing data.

Category	Reads (M)	Bases (Gb)	Depth(×)	Quality
WGS	1179.61	176.94	111.99	Q20: 97.80%; Q30: 92.63%
PacBio	3.50	65.08	41.19	N50: 17.7 kb; Read Quality (median): Q29
Hi-C	1981.69	297.25	188.13	Rate of valid reads: 19.36%
RNA-seq	48.82	7.32	—	Q30: 95.64%

**Table 2:** Statistics of genome assembly.

Category	Number
Genome size (bp)	1,580,604,568
Number of contigs	690
Number of chromosome-scale sequences	17
Number of unplaced scaffolds	208
Contig N50 (bp)	8,997,515
Contig N90 (bp)	2,188,720
Scaffold N50 (bp)	94,813,358
GC content (%)	37.53
Number of genes	34,469
Average gene length (bp)	18,099
Genome BUSCO (BUSCO software)	C:95.4%[S:92.3%,D:3.1%],F:2.8%,M:1.8%,n:954
Genome BUSCO (Compleasm)	C:97.7%[S:95.0%,D:2.7%],F:0.5%,M:1.8%,n:954
Gene set BUSCO	C:95.1%[S:92.2%,D:2.9%],F:1.4%,M:3.5%,n:954
Gene set Completeness (OMArk)	S:85.02%,D:5.67%[U:5.63%,E:0.04%],M:9.31%

**Table 3:** Statistics of Hi-C scaffolding.

Sequences	Length (bp)	Percentage (%)
Chr1	146,589,590	9.27%
Chr2	124,475,635	7.88%
Chr3	117,253,393	7.42%
Chr4	109,078,867	6.90%
Chr5	108,087,861	6.84%
Chr6	99,954,114	6.32%
Chr7	94,813,358	6.00%
Chr8	91,685,629	5.80%
Chr9	90,763,334	5.74%
Chr10	85,656,653	5.42%
Chr11	85,464,422	5.41%
Chr12	83,750,935	5.30%
Chr13	82,696,292	5.23%
Chr14	71,132,798	4.50%
Chr15	69,758,089	4.41%
Chr16	55,131,958	3.49%
Chr17	55,039,280	3.48%
Total length	1,571,332,208	99.41%
Unplaced scaffolds	9,272,360	0.59%

**Table 4:** Statistics of transposable elements (TE) annotation.

TE Type	Number	Length (bp)	Percentage (%)
ClassI-Retrotransposon	1,632,680	799,565,220	50.59
ClassI-LINE	316,820	124,776,826	7.89
ClassI-SINE	10,264	576,859	0.04
ClassI-LTR	1,305,480	674,211,535	42.66
ClassII-DNA transposon	259,724	81,904,905	5.18
Unknown	3632	3,932,552	0.25
Total	1,895,920	885,402,677	56.02

**Table 5:** Statistics of gene functional annotation.

Category	Number	Percentage (%)
Total	34,469	—
Swissprot	17,879	51.87%
KEGG	19,832	57.54%
TrEMBL	24,907	72.26%
Interpro	18,166	52.70%
GO	13,220	38.35%
Overall	25,655	74.43%

**Table 6:** Statistics of ncRNA annotation.

Type		Copy number	Average length(bp)	Total length(bp)	Percentage (%) of genome
miRNA		21	74.48	1,564	0.0001
tRNA		13,337	73.2	976,310	0.0618
rRNA	rRNA	665	165.35	109,961	0.007
	18S	181	356.8	64,581	0.0041
	28S	39	142.1	5,542	0.0004
	5.8S	0	0	0	
	5S	445	89.52	39,838	0.0025
snRNA	snRNA	488	152.5	74,419	0.0047
	CD-box	15	107.33	1,610	0.0001
	HACA-box	8	229.75	1,838	0.0001
	splicing	465	152.63	70,971	0.0045

## Methods

### Sampling and sequencing

Individuals of *Indoaustriella scarlatoi* were collected from peri-mangrove sediment in Wenchang, China (19°24′44″ N, 110°44′50″ E). Samples were fixed using RNAlater (Thermo Fisher Scientific) and stored at −80 °C.

The muscle tissue of one individual was used to extract the total DNA for WGS and PacBio HiFi sequencing. Genomic DNA was extracted using QIAamp DNA Mini Kit (Qiagen). For WGS, Covaris E220 was used to fragment DNA, and DNA fragments around 200 bp were selected using AMPure XP beads (Beckman). Selected fragments were amplified for eight PCR cycles and sequenced on the DNBSEQ sequencing platform (BGI) in a paired-end 150 bp layout (Table 1). Long-read sequencing was performed on the PacBio Sequel II system (PacBio). After examining the DNA using Qubit (Thermo Fisher Scientific) and pulsed field electrophoresis system (BioRad), a 15-kb PacBio library was constructed by g-TUBE (Covaris) shearing, end-repair, and BluePippin (Sage Science) size selection. Two SMART cells were sequenced through circular consensus sequencing (CCS) mode (Table 1). For Hi-C library construction, cells dissociated from *I. scarlatoi*’s muscle tissue were crosslinked with 1% formaldehyde and 0.2 M glycine. The fixed powder was resuspended in nuclei isolation buffer and then incubated in 0.5% SDS for 10 min at 62 °C, and the nuclei were collected by centrifugation. The DNA in the nuclei was digested with *Mbo*I (NEB), and the overhang was filled and biotinylated prior to ligation by T4 DNA ligase (NEB). After purification, DNA was sheared, and biotin-containing fragments were captured using Dynabeads MyOne Streptavidin T1 (Invitrogen). The captured DNA was then amplified and sequenced with NovaSeq 6000 (Illumina) with a layout of paired-end 150 bp (Table 1). To better annotate the genome assembly, RNA-seq of tissues from a whole clam was performed. Total RNA was extracted with TRIzol (Invitrogen) and used to generate cDNA with HiscriptII (Vazyme). The cDNA fragments were sequenced on the DNBSEQ platform, and 7.32 Gb 150 bp paired-end data was generated.

### Genome assembly and Hi-C scaffolding

Genome survey was conducted with WGS data using Jellyfish v2.2.6^15^ at K-mer 17, and the estimated genome size of *I. scarlatoi* was 1.48 Gb while the heterozygosity was 1.69%. The genome was assembled with PacBio data by hifiasm v0.16.1 (-k 45 -r 2 -a 2 -m 2,000,000 -p 20,000 -l 0)^16^. After that, the PacBio long-reads was realigned to the assembly using minimap2 v2.14^17^, and duplications in the assembly were removed using Purge_Dups v1.2.3 (https://github.com/dfguan/purge_dups) with default parameters. Kraken2^18^ was used to identify potential contaminant contigs, and contigs assigned to Bacteria were removed. The decontaminated contig-level assembly was assessed using BUSCO v5.2.2^19^ with metazoan odb10 (Table 2). The quality control of Hi-C data was performed using HiC-Pro v3.2^20^ (Table 1), and assembled contigs was then scaffolded by 3D-DNA^21^. Assembled chromosomes were visualized and adjusted in Juicebox v1.9^22^, and 99.41% of the contigs were anchored to 17 chromosomes (Table 3, Fig. 1A). The final assembly is 1.58 Gb with a scaffold N50 length of 94.81 Mb (Table 2, Fig. 1B).

### Repeat and gene annotation

Tandem repeats were annotated using Tandem Repeats Finder v4.0.7 with MaxPeriod set as 2000^23^. Transposable elements (TEs) were identified with both homology-based and d*e novo* prediction methods. LTR_Finder v1.0.6^24^ with parameters “-C” and RepeatModeler v1.0.8^25^ with default parameters were used for *de novo* search. For homology-based search, RepeatMasker v4.0.6^26^ was employed to search against Repbase v21.01^27^ with parameters “-nolow -norna -no_is” and results of *de novo* search (Table 4).

*Ab initio*, homology-based and gene expression evidence were combined to annotate protein-coding genes. Augustus v3.1^28^ was used for *ab initio* gene prediction. Blast v2.2.26^29^ was used to align gene sets from 10 molluscan species (*Archivesica marissinica*^6^, *Argopecten concentricus*^30^, *Conchocele bisecta*^7^, *Crassostrea gigas*^31^, *Gigantidas platifrons*^5^, *Lutraria rhynchaena*^32^, *Mactra quadrangularis*^33^, *Margaritifera margaritifera*^34^, *Modiolus philippinarum*^5^, *Pecten maximus*^35^) onto the genome of *I. scarlatoi*, and the alignment hits were linked to candidate gene region by GenBlastA^36^. GeneWise v2.2.0^37^ was employed to determine gene models with sequences of the candidate gene and their 2-kb flanking regions. RNA-seq data were mapped to the genome assembly by HISAT v2.1.0^38^, and Stringtie v1.3.4^39^ and Transdecoder v5.7.1 (github.com/TransDecoder/TransDecoder) with parameters “--complete_orfs_only” were used to generate the gene annotation with transcripts evidence. EVM v1.1.1^40^ was employed to integrate the results generated by the three methods with parameters “--segmentSize 5000000 --overlapSize 200000”, and the weights for integrating were “AUGUSTUS 1, GeneWise 3, transdecoder 10”. All annotated protein-coding genes were searched against the following databases: Swiss-Prot v201709, KEGG v87.0, InterPro v55.0, and TrEMBL v201709 (Table 5). Completeness of the gene set was assessed using BUSCO v5.2.2^19^ (Table 2).

ncRNA (non-coding RNA), including tRNA, rRNA, snRNA, and miRNA were predicted. tRNAscan-SE-1.3.1^41^ were used to predict tRNAs in the assembly with default parameters. We aligned invertebrate rRNA sequences against the assembly using BLAST software^29^ with “-e 1e-5”. For miRNA and snRNA annotation, we first aligned the assembly against the Rfam database^42^ (v14.1) using BLAST software^29^ (-e 1) to find candidate alignment, and used INFERNAL^43^ v1.1.1 to annotate snRNAs and miRNAs with default parameters (Table 6).

## Data Records

All sequencing data, including WGS, PacBio, Hi-C, RNA-seq, as well as the assembly (JBIWQA000000000)^44^ have been deposited at the NCBI (National Centre for Biotechnology Information) repository under project PRJNA1181275, SRP543674^45^. Genome assemblies and annotations of *I. scarlatoi* are also available at Figshare^46^.

## Technical Validation

The lengths of DNA fragments for PacBio sequencing mainly distributed around 50 kb, and the N50 length of PacBio reads is 17.7 kb. The size of the assembly is 1.58 Gb, while the estimated genome size by Jellyfish is 1.49 Gb. The quality value of the assembly, calculated using Merqury v1.3^47^, was 63.66, indicating high assembly accuracy. The assembled genome contains 690 contigs, which N50 length is 9.0 Mb and N90 is 2.2 Mb. The rate of valid Hi-C reads was 19.36%. After Hi-C scaffolding, 99.41% of the contigs were successfully anchored to 17 chromosomes. BWA (v0.7.17, github.com/lh3/bwa) MEM algorithm was used to align the WGS reads to the final assembly, and the mapping rate was calculated using the flagstat commands of samtools v1.9^48^ with the secondary mapping records removed. The mapping rate of WGS reads was 98.15%. In addition, we aligned RNA-seq data and PacBio HiFi reads against the assembly using hisat2^38^ and minimap2^17^ (“-ax map-hifi”), respectively, and the mapping rates of RNA-seq data were 81.15% while that of the HiFi reads was 99.78%. Using BUSCO software (v5.2.2)^19^, 938 of 954 BUSCOs were identified in the genome, including 911 complete ones, and the completeness of the final assembly was estimated as 95.4%. Compleasm v0.2.6^49^ was also used to test the completeness of the assembly and the result was 97.7% (Table 2). We used both BUSCO v5.2.2^19^ and OMArk v0.3.0^50^ to evaluate the quality of gene annotation, and the BUSCO score of gene set (95.1%) was similar with that of the assembly, while the OMArk completeness was 90.69%.

## Data Availability

Custom scripts for the Circos plot have been deposited at Git-hub (github.com/GuoYang-qd/Circos).
